# Extensive pityriasis versicolor presenting with truncal hypopigmentation: a rare clinical image

**DOI:** 10.11604/pamj.2026.53.23.50842

**Published:** 2026-01-16

**Authors:** Aarti Gopal Kute, Amol Deshpande

**Affiliations:** 1 Department of Rachana Sharir, Mahatma Gandhi Ayurved College Hospital and Research Centre, Datta Meghe Institute of Higher Education and Research (Deemed to be University), Salod (H), Wardha, Maharashtra, India

**Keywords:** Pityriasis versicolor, tinea versicolor, hypopigmented patches, *Malassezia* infection, truncal dermatoses

## Image in medicine

A 7-year-old male child presented to the dermatology clinic with no significant past medical history. There was no history of systemic illness, drug intake, or similar complaints among family members. The child complained of generalized itching for the past 9 months, with a noticeable increase in severity over the last month. Cutaneous examination revealed multiple ill-defined hypopigmented to light-brown macules and patches predominantly involving the back and anterior trunk. The lesions were irregular, coalescent, and formed a characteristic map-like pattern. Mild pruritus was present, while erythema and scaling were minimal. Diagnostic approach: the diagnosis was primarily clinical, based on the characteristic morphology, color variation, and truncal distribution of the lesions. The chronic course, minimal inflammation, and absence of systemic symptoms supported a superficial fungal etiology. The clinical presentation was consistent with pityriasis versicolor, commonly seen in children in warm and humid climates. The condition was managed as pityriasis versicolor, a superficial fungal infection caused by *Malassezia* species. Standard antifungal therapy (topical antifungals) was advised along with patient and caregiver counseling regarding the benign nature of the disease and the possibility of recurrence.

**Figure 1:**
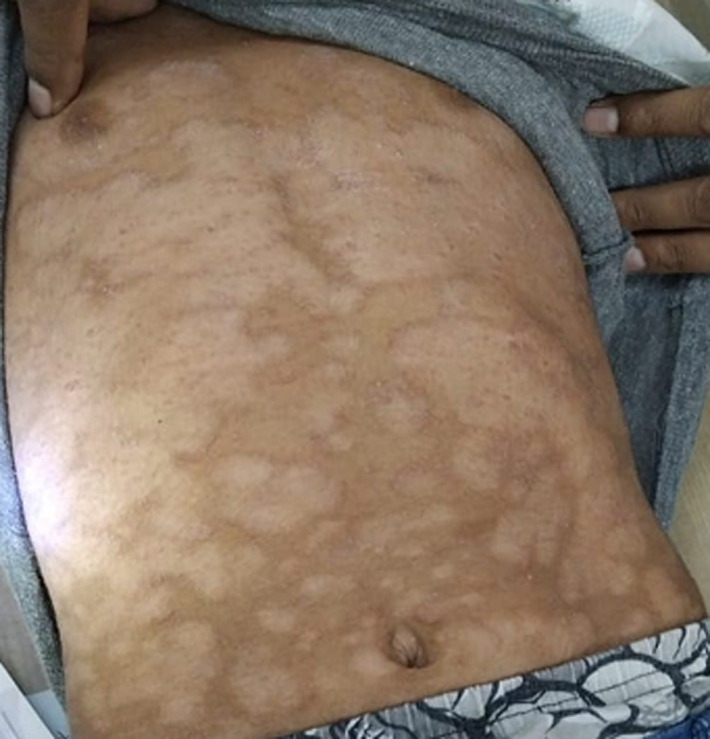
multiple hypopigmented, irregular, coalescent patches over the abdomen consistent with pityriasis versicolor

